# MnEdgeNet for accurate decomposition of mixed oxidation states for Mn XAS and EELS L2,3 edges without reference and calibration

**DOI:** 10.1038/s41598-023-40616-5

**Published:** 2023-08-29

**Authors:** Zhengran Ji, Mike Hu, Huolin L. Xin

**Affiliations:** Department of Physics and Astronomy, University of California, Irvine, Irvine, CA 92697 USA

**Keywords:** Characterization and analytical techniques, Microscopy

## Abstract

Accurate decomposition of the mixed Mn oxidation states is highly important for characterizing the electronic structures, charge transfer and redox centers for electronic, and electrocatalytic and energy storage materials that contain Mn. Electron energy loss spectroscopy (EELS) and soft X-ray absorption spectroscopy (XAS) measurements of the Mn L2,3 edges are widely used for this purpose. To date, although the measurements of the Mn L2,3 edges are straightforward given the sample is prepared properly, an accurate decomposition of the mix valence states of Mn remains non-trivial. For both EELS and XAS, 2+, 3+, and 4+ reference spectra need to be taken on the same instrument/beamline and preferably in the same experimental session because the instrumental resolution and the energy axis offset could vary from one session to another. To circumvent this hurdle, in this study, we adopted a deep learning approach and developed a calibration-free and reference-free method to decompose the oxidation state of Mn L2,3 edges for both EELS and XAS. A deep learning regression model is trained to accurately predict the composition of the mix valence state of Mn. To synthesize physics-informed and ground-truth labeled training datasets, we created a forward model that takes into account plural scattering, instrumentation broadening, noise, and energy axis offset. With that, we created a 1.2 million-spectrum database with 1-by-3 oxidation state composition ground truth vectors. The library includes a sufficient variety of data including both EELS and XAS spectra. By training on this large database, our convolutional neural network achieves 85% accuracy on the validation dataset. We tested the model and found it is robust against noise (down to PSNR of 10) and plural scattering (up to t/λ = 1). We further validated the model against spectral data that were not used in training. In particular, the model shows high accuracy and high sensitivity for the decomposition of Mn_3_O_4_, MnO, Mn_2_O_3_, and MnO_2_. The accurate decomposition of Mn_3_O_4_ experimental data shows the model is quantitatively correct and can be deployed for real experimental data. Our model will not only be a valuable tool to researchers and material scientists but also can assist experienced electron microscopists and synchrotron scientists in the automated analysis of Mn L edge data.

## Introduction

X-ray absorption spectroscopy (XAS)^1^ and electron energy loss spectroscopy (EELS)^2, 3^ are two techniques that can probe the unoccupied electronic states providing bonding information of materials. In particular, the L2,3 edges are widely used to determine the oxidation state of transition metals^1, 4, 5^. The transition metal L2,3 edges probe the unoccupied *d* orbitals and therefore the edge onset and the edges’ fine structures and shapes are sensitive to the oxidation state of the *d*-block metal ions, in particular the 3*d* transition metals, such as V, Ti, Mn, Fe, and Ni^5–8^. For example, using the near-edge fine structures in the Mn L2,3 edges, the oxidation states of Mn ions in a material can be determined by decomposing the spectrum into a linear combination of Mn2+, Mn3+, and Mn4+ reference spectra^9, 10^. This decomposition, in principle, is simple but in reality, it is non-trivial because the energy axis is not always calibrated, and the instrument/beamline does not always have the instrumental broadening. Without proper calibration, an energy offset is present between the experimental spectrum and the references which prevents accurate oxidation state decomposition. In order to avoid the problem, standard reference samples such as MnO, Mn_2_O_3_, and MnO_2_ need to be measured in the same experimental session to avoid any energy offsets as well as changes in instrumental broadening^9, 11^. Still, with this procedure, other factors could prevent the proper energy axis calibration, for example, temperature fluctuations would result in an energy shift in the monochromator for XAS experiments. Basically, if the XAS measurements are separated multiple hours in time, the spectra taken could have a slight energy offset. In EELS, the energy offset could change more rapidly and is more unpredictable than in XAS. Typically, the energy offset is very sensitive to the DC stray field. For example, the passing of a heavy-duty truck or the movement of a nearby elevator could change the energy offset if the TEM instrument is not fully shielded. This problem is now mitigated with the dualEELS instruments but there are still many single EELS instruments under active service. Moreover, all historical data were acquired without the dualEELS correction. In addition, the nonlinearity of the parallel EELS spectrometer is present in EELS in a nontrivial way because the nonlinearity is not only present in the dispersion device, i.e., the magnetic prism. There is another complex nonlinearity present in the magnification lenses, a series of quadrupoles. Therefore, it is extremely difficult to calibrate the energy onset of EELS edges unless strict protocols are followed as described by Tan et al.^11^.

Another complication is that EELS’ near-edge fine structures change with sample thickness due to plural scattering. As the sample gets thicker, signals close to the edge onset would be multiply scattered to higher energy losses. This would result in a shape change of the spectrum^11^. For example, for the latter 3*d* transition metals’ L2,3 edges, as the sample gets thicker, the L2/L3 ratio increases—this problem has rendered the reference-free L2,3 ratio method inaccurate for EELS^11^. In addition, for XAS, the background and the near-edge structures could be different between the Total Electron Yield (TEY) and Partial Fluorescence Yield (PFY) modes. TEY mode measures the total number of emitted electrons resulting from the absorption of X-rays while PFY measures the fluorescence emitted by the sample as a result of X-ray absorption. That also renders the L2,3 ratio method unreliable. Moreover, for early 3*d* transition metals, there are no established reference-free methods because of the L2,3 anomaly.

For both EELS and XAS spectroscopy, one interesting observation is that human operators with sufficient training can identify spectral features and assign oxidation states to transition metal L2,3 edges with high confidence. This points to the direction that deep learning could be successful in solving the L2,3 oxidation state decomposition problem. Pate et al. in 2021 discussed using deep learning to denoise high frame rate spectra^12^. Chatzidakis and Botton in 2019 introduced the idea of translation-invariance for classifying EELS edges^13^. They built a convolutional neural network (CNN) for oxidation state classification and showed that with translation-invariant training, moving the energy axis does not change the Mn2+, 3+, and 4+ oxidation state classification. This is a very important step in demonstrating that spectral features are like spatial features in images—they can be classified by a CNN network regardless of their absolute energy positions in the spectrum. However, there are still problems remained to be resolved: (1) how to quantitatively decompose mixed oxidation states; (2) is it possible to build one model that works for both XAS and EELS spectroscopy that have drastically different energy resolutions; (3) is it possible to build a model that is not affected by plural scattering, i.e., the thickness effect in EELS.

To address the three challenges defined above, in this study, we present a reference-free, calibration-free deep learning approach to determine the accurate oxidation states decomposition of 3*d* transition metal based on the L2,3 near-edge fine structures. To demonstrate the validity of the method, we use Mn as an example because Mn is technologically important in catalysis, energy storage, and electronic materials. Also, Mn oxides are a good case study because their 3 different oxidation states lead to notable variations in fine structures of the Mn L2,3 edge. Determining the composition of the mixed oxidation states is extremely important for understanding the charge transfer phenomenon happening at the device interfaces. The method we present in this study is not a simple classification of Mn2+, 3+, and 4+ edges but an accurate and quantitative decomposition of the mixed Mn oxidation states. Instead of having a classification/binary type label, we created a three-element ground truth vector that quantitatively describes the composition of Mn2+, 3+, and 4+ in each Mn spectrum, i.e. [%Mn_2+_, %Mn_3+_, %Mn_4+_].

To achieve this goal, we synthesized a spectrum library from 38 experimental spectra (23 EELS and 15 XAS). The library contains 1.2 million spectra 50% of which are synthesized from XAS data and the other 50% are synthesized from EELS data. In building the mixed oxidation state library, we paid special attention to normalizing the Mn L2,3 edges correctly, and including experimental-like uncertainties such as both Gaussian and Lorentzian type instrumental broadening, energy offset, and detector noise. To include the plural scattering effect in the training library, we developed a forward model to correctly introduce the thickness effect to the L2,3 edges. Using this physics-informed large training library, we show that the deep convolutional regression model we trained is robust against plural scattering and noise. The overall accuracy of the model in determining the mixed valence state reaches 85% on the validation data set. We also validated the data on “unknown unknowns”, i.e., Mn_3_O_4_ spectra that have never been used for training and validation—the accurate decomposition of Mn_3_O_4_ experimental data shows the model is quantitatively correct and can be deployed for real experimental data.

## Methods

In this method section, we will describe (1) how to build a ground-truth oxidation state labeled Mn edge library, (2) how to construct the neural network, and (3) how to train it.

For building the library, the technical challenges lie in (1) how to obtain a wide variety of XAS and EELS Mn2+, Mn3+, and Mn4+ reference spectra; (2) how to normalize or ratio the 2+, 3+, and 4+ spectra correctly; (3) how to include the EELS’s plural scattering effect (thickness effect) into the training sets; (4) how to include the various experimental uncertainties including instrumental broadening, energy offset, detector noise, etc. In the following subsections, we will address each aforementioned challenge.

### Collection of Mn reference spectra

**Figure 1:**
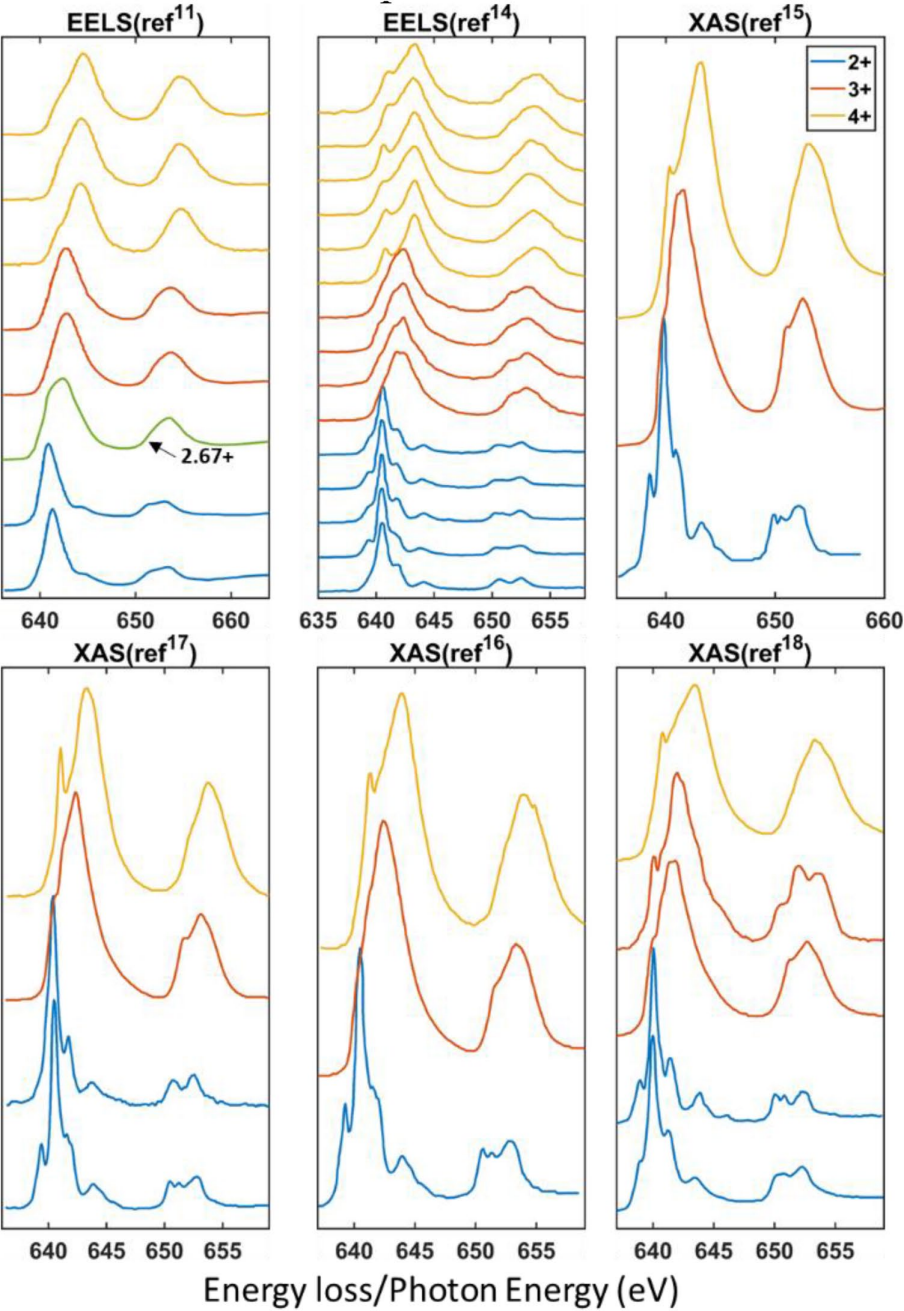
The presentation of the EELS and XAS Mn L2,3 edges included in making the training library. The Mn 2.67+ presented is not included in the training library.

To have sufficient varieties of data that can capture the features of the EELS and XAS Mn 2+, 3+, and 4+ edges, in this study, we digitized 23 experimental EELS and 13 XAS Mn spectra, in total 38, that were documented in 6 literatures using WebPlotDigitizer^20^. In Fig. 1, we presented all spectra that were used for making the training library. (The Mn 2.67+ spectrum was not included in the training library). In Table 1, we listed the compounds for which we digitized the spectra and their original references.

**Table 1:** The compound information and references of the Mn L2,3 edges.

Oxidation state	Compounds	References
2+	MnO (Manganosite), MnV2O4, (LiMnPO_4_) Lithiophilite, (MnSiO_3_) Rhodonite, MnF2, (MnCO3) Rhodochrosite, YBaMn3AlO7,	EELS Refs.^11, 14^ XAS Refs.^15–18^
3+	MnOOH(Manganite), Mn_2_O_3_, ((Mn,Fe)_2_O_3_) Bixbyite, (Ca_4_Mn^3+^_2–3_(BO_3_)_3_(CO_3_)(O,OH)_3_) Gaudefroyite, LaMnO3	
4+	SrMnO3, CaMnO3, MnO_2_, Todorokite, ((Ni,Co)_2-x_Mn^4+^(O,OH)_4_ · nH_2_O) Asbolan, (ZnMn^4+^_3_O_7_ · 3H_2_O)Chalcophanite, (Mn^4+^O_2_) Ramsdellite, (Mn^4+^O_2_) Pyrolusite	
2.67+	Mn_3_O_4_ (not used for training)	

All data are standardized to range from 630.5 eV to 669.4 eV with 0.1 eV increments (338 data points). For missing data, the left side of the spectra is padded with zero and the right side is padded with the end value of the spectra.

### Normalization of 2+, 3+, 4+ reference spectra

**Figure 2:**
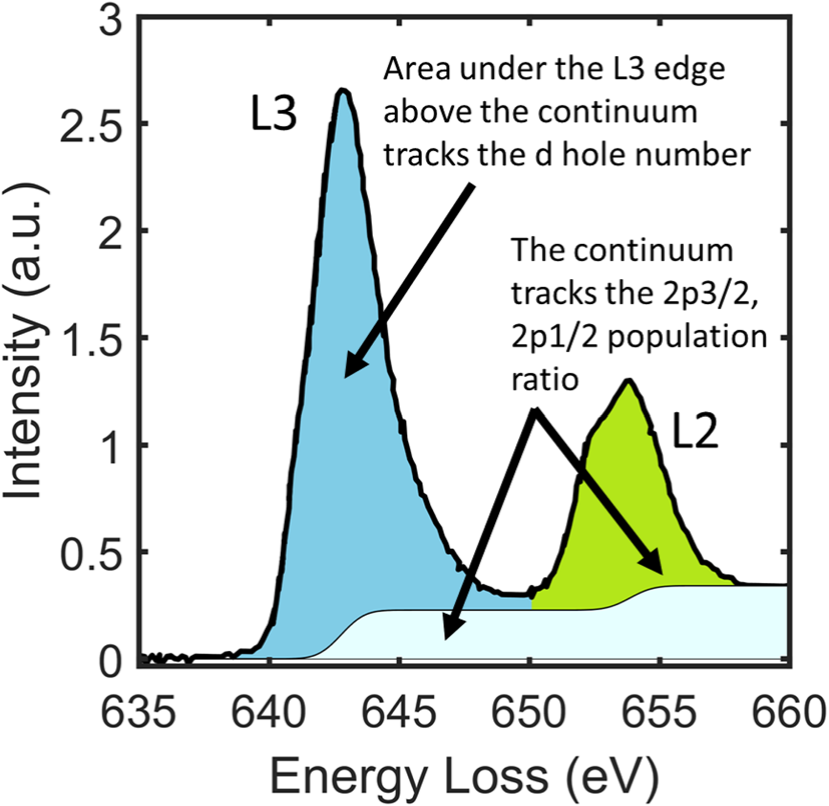
Schematics showing how to extract the *d*-hole area under the L3 edge.

In order to quantitatively combine the 2+, 3+, and 4+ Mn spectra, they need to be normalized to the correct ratio. To achieve that, we normalize the Mn L3 edge according to the *d*-hole number. Elemental Mn has an electron configuration of [Ar] 3*d*5 4*s*2. Therefore, Mn2+, 3+, 4+ have an electron configuration of [Ar] 3*d*5, [Ar] 3*d*4, [Ar] 3*d*3. Because the *d* shell can hold 10 electrons, the number of holes for Mn 2+, 3+, and 4+ are 5, 6, and 7 respectively. Therefore, the area under the L3 peak and above the continuous background shall be proportional to the *d-*hole number. The continuous background under the L2,3 edge can be modeled by two-step functions with a step height that follows the 1:2 population ratio. (The filled 2*p*3/2, and 2*p*1/2 orbitals have a population ratio of 1:2). The *d*-hole area can be calculated after the background is subtracted from the spectrum (Fig. 2). With this procedure to find the *d*-hole area, we can correctly ratio the 2+, 3+, and 4+ spectra.

### Ground truth labeled library

After the *d*-hole ratio normalization, we can correctly combine the Mn 2+, 3+, and 4+ component spectra to form a new spectrum with the known ground truth oxidation state composition and oxidation state as the following

$$\begin{aligned} & s = xMn^{2 + } + yMn^{3 + } + zMn^{4 + } \\ & where\,x + y + z = 1 \\ & {\text{ground}}\;{\text{truth}}\;{\text{oxidation}}\;{\text{state}}\;{\text{composition }} = \, \left[ {x,y,z} \right] \\ & {\text{ground}}\;{\text{truth}}\;{\text{average}}\;{\text{oxidation}}\;{\text{state }} = { 2}x + { 3}y + { 4}z. \\ \end{aligned}$$

In making the ground truth labeled spectra, we only combined Mn spectral components that were digitalized from the same publication source. The reason is the spectra to be combined shall share the same instrumental resolution.

The composition of the training library is detailed in Table 2. A total of 1,200,000 synthetic spectra are included in the library.

**Table 2:** The composition of the ground truth labeled library.

Components	Occurrence	Type
Single component 2+, 3+, or 4+	2.5%	50% EELS and 50% XAS
Two components (2+, 3 +), (2+, 4+), or (3+, 4+)	48.75%	50% EELS and 50% XAS
Three components (2+, 3+, 4+)	48.75%	50% EELS and 50% XAS

### Instrumental broadening

The instrumental broadening of EELS spectra includes two major contributions. The first contribution primarily comes from the thermal broadening at the electron source and the first crossover due to the space-charge effect. This type of broadening is typically characterized by a Gaussian type broadening function. The second contribution happens at the detector. The light diffusion in the sinterlator and the optical coupler introduce a long-tail broadening effect which can be characterized by a Lorentzian function. For XAS, similar short-range and long-range broadening happens due to the monochromator. Therefore, we introduce a two-parameter controlled instrument broadening kernel aka the point spread function, PSF(E) as the following.

$${\text{PSF}}\left( {E,w,\sigma } \right) = {\text{L}}\left( {E,w} \right) \otimes {\text{G}}\left( {E,\sigma } \right)$$

where ⊗ stands for convolution

$$L(E,w) = \frac{1}{\pi }\frac{1/2w}{{E}^{2}+(1/2w{)}^{2}}$$

and

$$G(E,\sigma ) = \frac{1}{\sigma \sqrt{2\pi }}{e}^{-\frac{1}{2}(\frac{E}{\sigma \sqrt{2}})}$$

**Figure 3:**
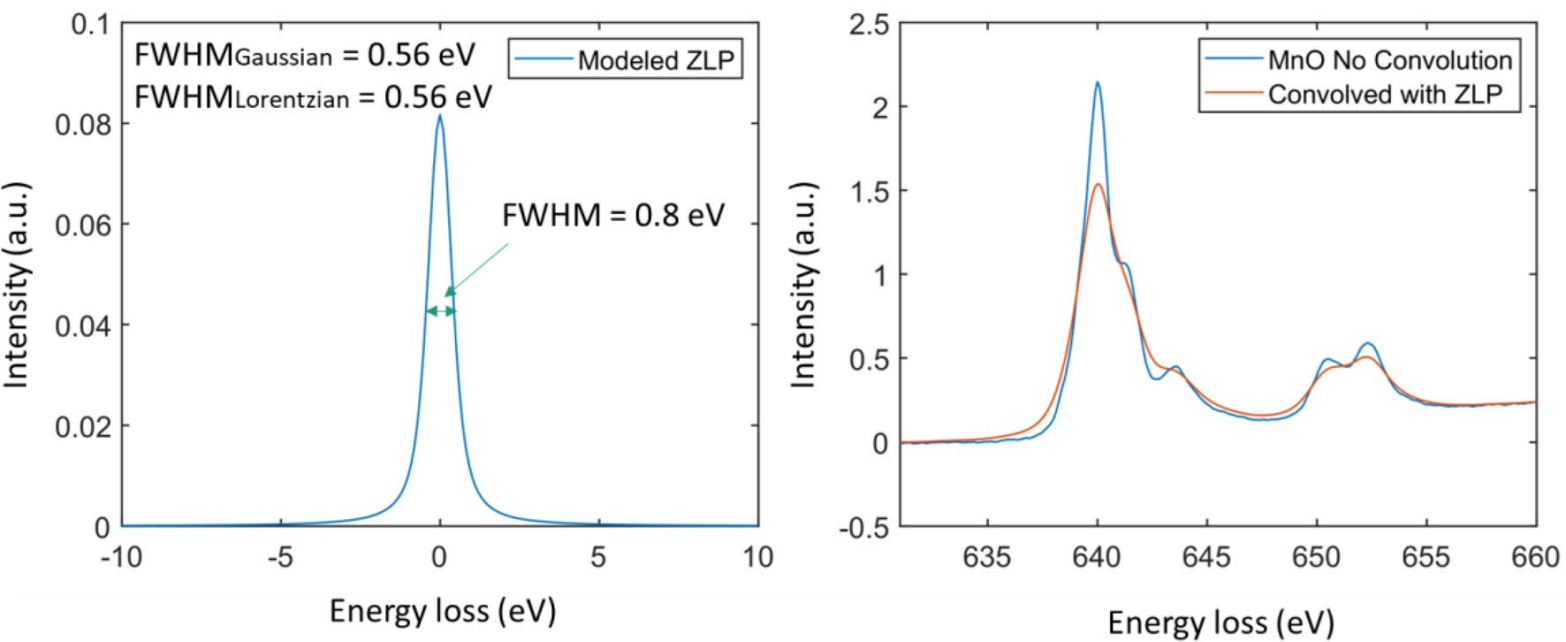
An example of the instrumental broadening effect on the Mn L2,3 edge.

Basically, the instrumental point spread function is a convolution of a Gaussian function with a Lorentzian function. The full width at maximum (FWHM) of the Lorentzian function is *w* and the FWHM of the Gaussian function is $2\sqrt{2ln(2)}\sigma$. The combined FWHM is equal to $\sqrt{FWH{M}_{Lorentzian}+FWH{M}_{Gaussian}}$. It is worth noting that the inclusion of the Lorentzian tails in the point spread kernel is very important for making the synthesized spectra resemble the experimental ones. An example of such a broadening effect on a Mn2 + L2,3 edge is shown in Fig. 3.

### Plural scattering in EELS

If the single scattering probability function is *P*(E), plural scattering as a function of thickness, *t*, in EELS can be described by the following differential equation

$$\frac{dS(E, t)}{dt}={\int }S(E{\prime})P(E-E{\prime})dE{\prime}$$

and the boundary condition is $S(E,t=0) = \delta (E)$ in the ideally monochromatic condition. In the practical situation where the incoming electron has an energy spread, we can use the point spread function given in the last section as the initial energy profile, i.e.

$$S(E,t=0) = PSE(E,w,\sigma )$$

Once we obtain a numerical representation of *P*(*E*), the spectral function, *S*(*E*, *t*) at any given thickness, *t* can be numerically calculated.

Using this equation, it allows us to calculate the low-loss spectral function numerically. Once we obtain the low-loss spectral function, the core-loss spectrum is a convolution of the core-loss single scattering probably distribution, ${P}_{core-loss}(E)$, with the low-loss spectral function, i.e., $S(E,t)$.

**Figure 4:**
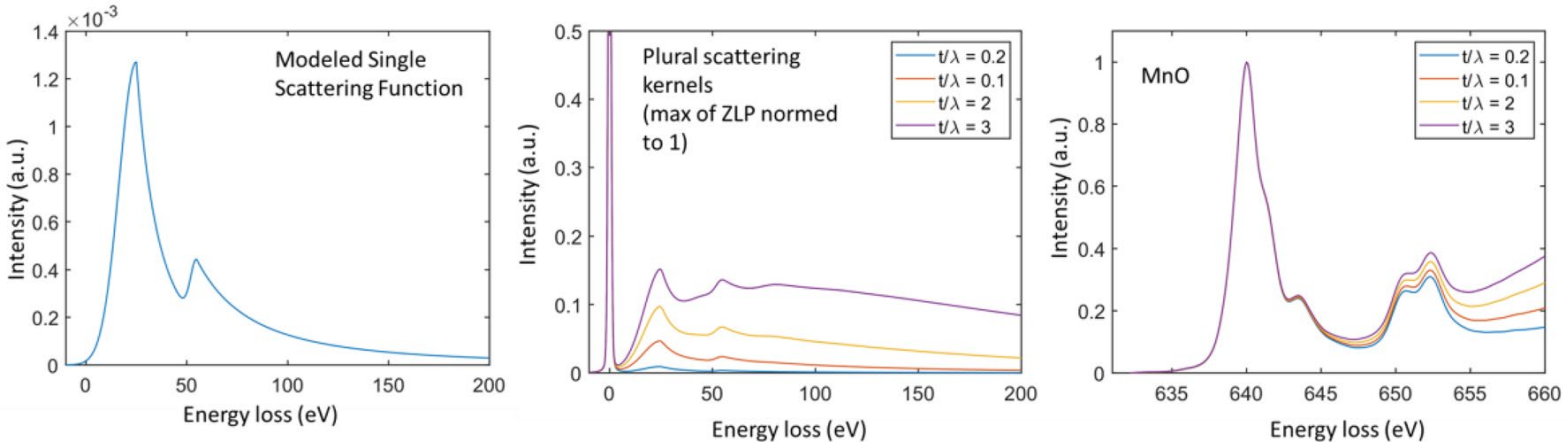
The modeling of the plural scattering for Mn-containing compound and its effect on the spectral shape of Mn L2,3 edges.

Figure 4 shows the change of the low-loss function as a function of normalized thickness ($t/\lambda$, $\lambda$ is the inelastic mean free path) and how the Mn L2,3 edge evolves.

In this modeling, we use an average plasmon loss energy of 25 eV and approximate the *P*(*E*) by an asymmetric function where the left side is a Gaussian function, and the right size is a Lorentzian loss function. To be more exact, we also modeled the Mn M edge and superimposed it onto the plasmon loss.

### Other augmentations: energy shift and noise

Both EELS and XAS are subject to the issues of inaccurate energy axis. To take this into account, we apply a random shift augmentation of the energy axis for the ground truth labeled spectra. With this augmentation, the model becomes translation invariant—it is only sensitive to the spectral shape and it is insensitive to the absolute energy onset of the L2,3 edge.

For noise, we have modeled the noise as white noise (Gaussian noise) with a salt and pepper noise (impulse noise). Both noises are additive to the spectrum. We use the linear definition of PSNR as:

$$PSNR=\frac{Max\,Signal}{\sqrt{Mean\,Sequare\,Error}}$$

## Summary of augmentation

In Table 3, we summarize the augmentation operations done to the ground truth labeled library.

**Table 3:** A summary of the augmentation operations and occurrences.

Type of augmentation	Probability of application	Parameters
Instrumentation broadening	80%	Gaussian: FWHM uniformly distributed between 0.01 and 1.5 eV Lorentzian: FWHM uniformly distributed between 0.1 and 0.4 eV
Plural scattering	80%	Normalized thickness $t/\lambda$ uniformly distributed between 0 and 1
Shifts	100%	Uniformly distributed between -4 eV and + 4 eV
Noise	50%	PSNR ranges from 10 to 30

### Network structure

**Figure 5:**
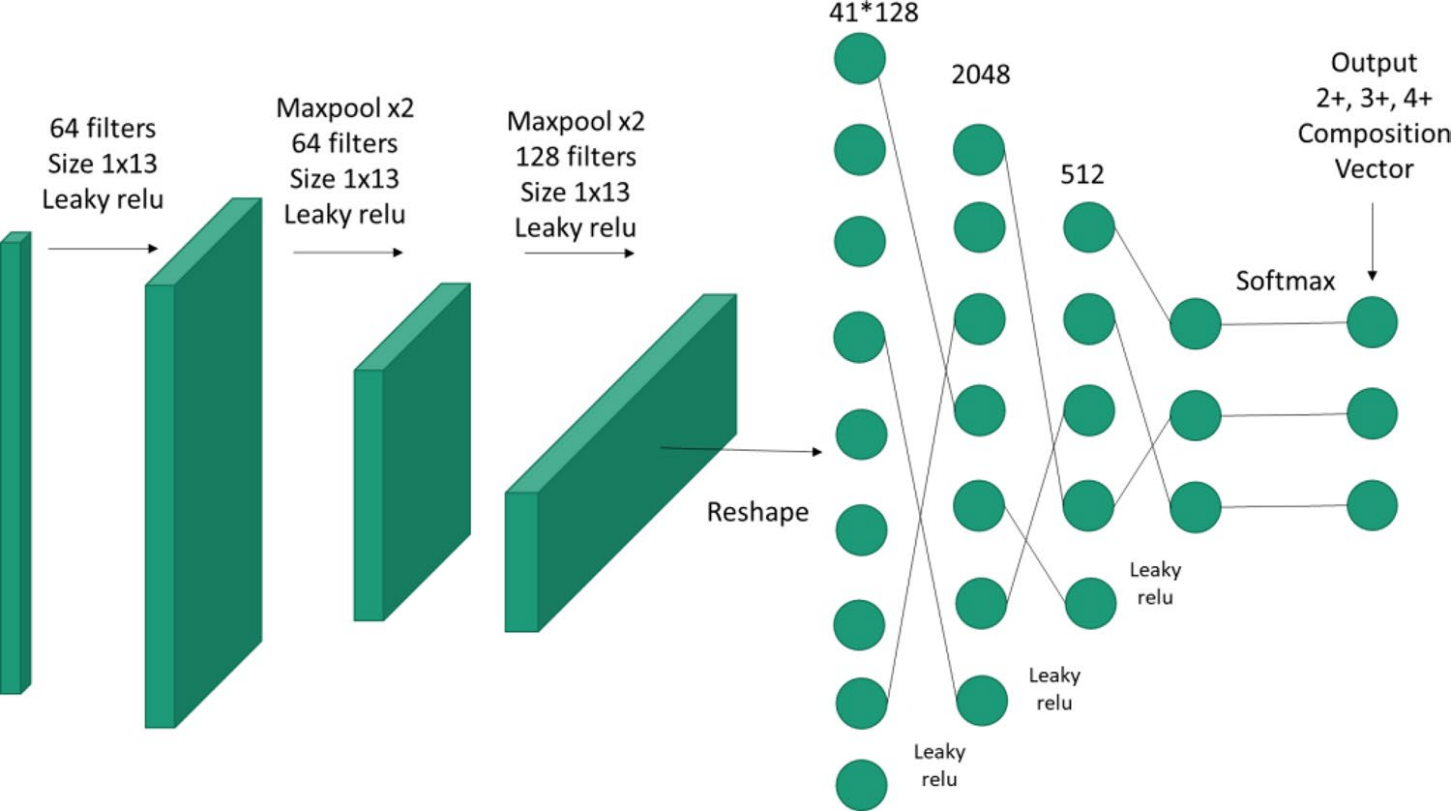
The structure of the convolutional regression net for mixed oxidation state decomposition.

How our brains process or identify a spectral feature is very similar to recognizing spatial features in an image. Inspired by this, we adopted the convolution layers that are used in image classification for feature extraction. Then we connected the features with a fully connected layer (also known as dense layers) for composition regression. The input is the one-dimensional spectrum, and the output is a 3-element composition vector (Fig. 5). We call this network a convolutional regression net (CRN). Different from a classification network, a regression network’s outputs are continuous numbers rather than binary numbers. Therefore, we used the mean square error function as the loss function.

For feature extractions, we use three convolutional layers followed by leaky ReLU and maxpooling. The final layer outputs 41*128 = 5248 filtered features. In the regression layers, we used three fully connected layers with 2048, 512, and then 3 neurons with leaky ReLU in between. The final output is a softmax normalization of the final 3-neuron layer to ensure that the sum of the composition vector is equal to 1.

### Training

In Table 4, we summarized the technical information of the training process.

**Table 4:** Technical information for training.

Parameter	Value
Framework	PyTorch
GPU	NVIDIA GeForce GTX 1080 Ti
Training time	3.53 h
Optimizer	Adaptive moment estimation (Adam)
Learning rate	0.00008
Loss function	Mean squared error loss (MSELoss)
Max Epochs	4
Batch Size	32

**Figure 6:**
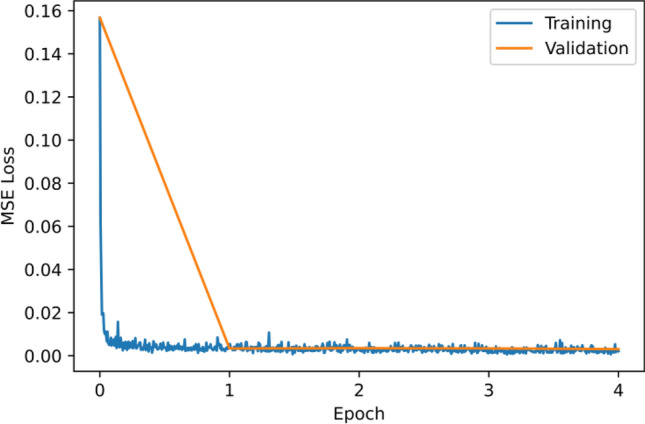
The MSE loss as a function of epochs processed.

All spectra are subtracted by the mean and divided by the standard deviation before entering the network. Dropouts are added to each layer before maxpooling with a dropout rate of 0.1. Adam, an algorithm for first-order gradient-based optimization of stochastic objective functions, based on adaptive estimates of lower-order moments was used for learning. The learning rate is set at 8E-5. The batch size is 32. As shown in Fig. 6, the model converges quickly; therefore, only 4 epochs of training were used to avoid overfitting.

### Validation

We performed an 80/20 split of the ground truth labeled library to split it into 80% of training and 20% of validation datasets. The accuracy of the model is evaluated on the validation set. An accurate prediction is defined as the predicted oxidation state falling in the range of ± 0.1 of the ground truth oxidation state.

We also evaluated our model on reference data and testing data. Reference data are the data we digitized from the literature, used to build the library. The testing data are new experimental and literature data that were never used for the construction of the training data.

## Result and discussion

### Performance of the model on the validation set

**Figure 7:**
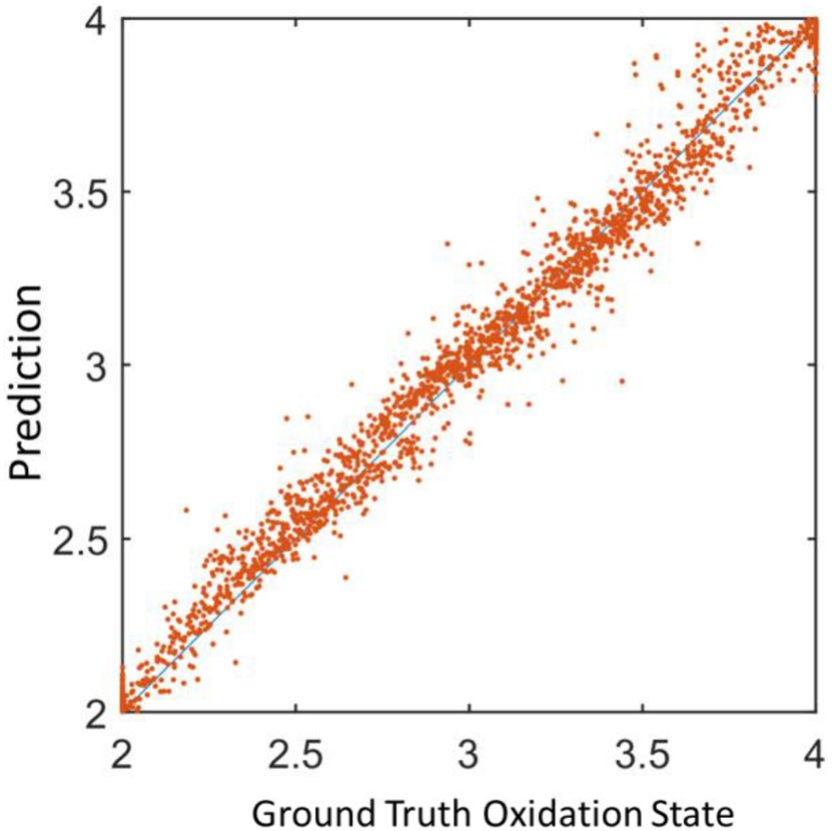
The scatter plot of the model’s predicted average oxidation state versus the ground truth.

On the validation set, the trained model reports an accuracy of 85%. Figure 7 shows the scatter plot of the prediction versus the ground truth (2000 spectra were randomly selected from the validation set). The result shows that the model performs reasonably well on the validation data.

To more closely look at the performance of the predicted decomposition, we provide a table of the predicted composition as shown in Table 5. It shows that the decomposition is reasonably accurate on the validation dataset.

**Table 5:** CRN’s decomposition performance on validation spectra.

Compound	Predicted [2+, 3+, 4+] decomposition (%)	Predicted oxidation state	Ground truth
Mn_2_O_3_	[0.98, 98.5, 0.52]	3.0	3.0
MnF2	[99.69, 0.31, 0.0]	2.0	2.0
MnF3	[0.66, 98.87, 0.48]	3.0	3.0
MnO	[98.05, 1.85, 0.1]	2.02	2.0
MnO_2_	[0.45, 4.67, 94.88]	3.94	4.0
MnO	[99.6, 0.37, 0.03]	2.0	2.0
MnV2O4	[99.42, 0.21, 0.37]	2.01	2.0
MnOOH	[0.18, 98.03, 1.79]	3.02	3.0
CaMnO3	[0.0, 2.4, 97.6]	3.98	4.0
MnO_2_	[0.0, 0.01, 99.98]	4.0	4.0
SrMnO3	[0.0, 0.06, 99.94]	4.0	4.0
75% Mn3 + OOH + 25%Mn4+ O2	[0.09, 73.45, 26.46]	3.26	3.25
75% Mn3 + OOH + 25% SrMn4+ O3	[0.25, 70.55, 29.2]	3.29	3.25
60% Mn4+ O2 + 40% SrMn4+ O3	[0.0, 0.02, 99.98]	4.0	4.0

### Plural scattering

To show how the model performs with the interference of plural scattering, we tested the thickness effect on MnO, Mn_2_O_3_, and MnO_2_. As shown in Table 6, the results are accurate up to 1.5 inelastic mean free path (λ) which is larger than the maximum augmentation range used in the training dataset.

**Table 6:** Testing of CRN’s decomposition robustness against plural scattering on validation data.

Thickness	Predicted decomposition (%)	Predicted oxidation state
*MnO*		
0	[99.6, 0.37, 0.03]	2.0
0.1	[99.49, 0.5, 0.01]	2.01
0.3	[99.41, 0.58, 0.01]	2.01
0.5	[99.28, 0.71, 0.01]	2.01
0.8	[98.94, 1.04, 0.02]	2.01
1	[98.64, 1.33, 0.03]	2.01
1.5	[97.28, 2.57, 0.16]	2.03
2	[94.27, 4.14, 1.59]	2.07
2.5	[88.33, 4.42, 7.25]	2.19
3	[80.74, 3.14, 16.12]	2.35
*Mn*_*2*_*O*_*3*_		
0	[0.66, 99.13, 0.21]	3.0
0.1	[0.57, 99.21, 0.22]	3.0
0.3	[0.6, 99.19, 0.21]	3.0
0.5	[0.69, 99.07, 0.24]	3.0
0.8	[1.25, 98.46, 0.28]	2.99
1	[2.2, 97.54, 0.26]	2.98
1.5	[12.87, 86.66, 0.48]	2.88
2	[43.15, 55.66, 1.19]	2.58
2.5	[52.78, 44.89, 2.33]	2.5
3	[59.93, 36.5, 3.57]	2.44
*MnO*_*2*_		
0	[0.0, 0.01, 99.98]	4.0
0.1	[0.0, 0.03, 99.97]	4.0
0.3	[0.01, 0.04, 99.95]	4.0
0.5	[0.01, 0.06, 99.93]	4.0
0.8	[0.01, 0.11, 99.88]	4.0
1	[0.02, 0.21, 99.77]	4.0
1.5	[0.11, 1.75, 98.14]	3.98
2	[0.57, 10.88, 88.55]	3.88
2.5	[3.33, 23.44, 73.23]	3.7
3	[16.82, 20.4, 62.78]	3.46

### Performance with noise

To show how the model performs with the interference of noise, we tested the effect on MnO, Mn_2_O_3_, and MnO_2_. As shown in Table 7, the model is robust down to PSNR = 20. At PSNR = 10, 2+ and 4+ are more stable than 3+.

**Table 7:** Testing of CRN’s decomposition robustness against noise on validation data.

PSNR	SNR	Predicted decomposition (%)	Predicted oxidation state
*MnO*			
None	None	[99.6, 0.37, 0.03]	2.0
PSNR = 30	SNR = 7.5	[99.97, 0.02, 0.01]	2.0
PSNR = 20	SNR = 5	[99.99, 0.0, 0.0]	2.0
PSNR = 10	SNR = 2	[99.68, 0.3, 0.03]	2.0
PSNR = 5	SNR = 1.25	[99.74, 0.26, 0.0]	2.0
PSNR = 3	SNR = 0.75	[99.95, 0.05, 0.0]	2.0
*Mn*_*2*_*O*_*3*_			
None	None	[0.25, 99.59, 0.16]	3.0
PSNR = 30	SNR = 7.5	[0.94, 98.08, 0.98]	3.0
PSNR = 20	SNR = 5	[1.58, 98.41, 0.01]	2.98
PSNR = 10	SNR = 2	[22.09, 77.91, 0.0]	2.78
PSNR = 5	SNR = 1.25	[89.7, 9.85, 0.44]	2.11
PSNR = 3	SNR = 0.75	[0.0, 0.0, 99.99]	4.0
*MnO*_*2*_			
None	None	[0.0, 0.01, 99.98]	4.0
PSNR = 30	SNR = 7.5	[0.0, 0.05, 99.95]	4.0
PSNR = 20	SNR = 5	[0.0, 0.0, 100.0]	4.0
PSNR = 10	SNR = 2	[0.09, 10.54, 89.37]	4.0
PSNR = 5	SNR = 1.25	[4.73, 0.13, 95.14]	3.89
PSNR = 3	SNR = 0.75	[0.0, 0.0, 100.0]	3.9

### Validation of the model on testing data

Validation of testing data is critical for understanding the accuracy and robustness of a machine learning model.

### Testing on Mn_3_O_4_

**Figure 8:**
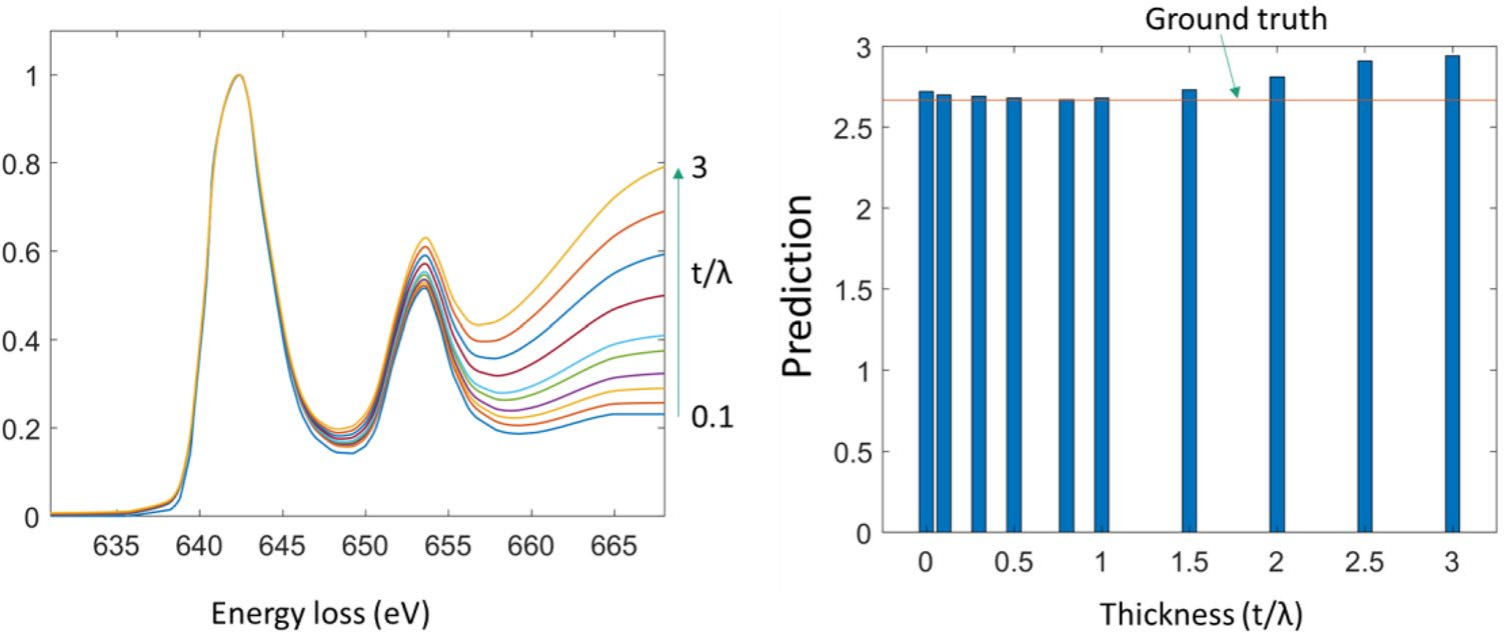
Validation on Mn_3_O_4_ as a function of thickness.

One of the compounds, for which we have experimental data on, but was not used for training was Mn_3_O_4_. It has a mixed oxidation state of Mn2+ and Mn3+ with a theoretical ratio of 1:2. It gives an average oxidation state of + 2.67. Figure 8 shows the predicted oxidation state as a function of thickness and the oxidation state decomposition is shown in Table 8. The model predicts the correct ratio between 2+/3+ with a small error. The prediction starts to deviate from the ground truth at t/λ = 1.5 which is larger than the maximal augmentation used for training. Therefore, reduced performance is expected.

**Table 8:** Testing of CRN’s decomposition robustness against plural scattering on testing data(Mn_3_O_4_).

Thickness	Predicted decomposition (%)	Predicted oxidation state
0.0	[32.03, 64.39, 3.59]	2.72
0.1	[32.00, 64.02, 2.99]	2.7
0.3	[33.81, 63.79, 2.4]	2.69
0.5	[34.31, 63.67, 2.02]	2.68
0.8	[34.39, 64.03, 1.59]	2.67
1.0	[34.16, 64.1, 1.74]	2.68
1.5	[32.28, 64.1, 1.74]	2.73
2.0	[30.24, 58.1, 11.67]	2.81
2.5	[27.73, 53,69, 18.57]	2.91
3.0	[28.38, 49.6, 22.02]	2.94

### Further testing on the influence of noise

**Figure 9:**
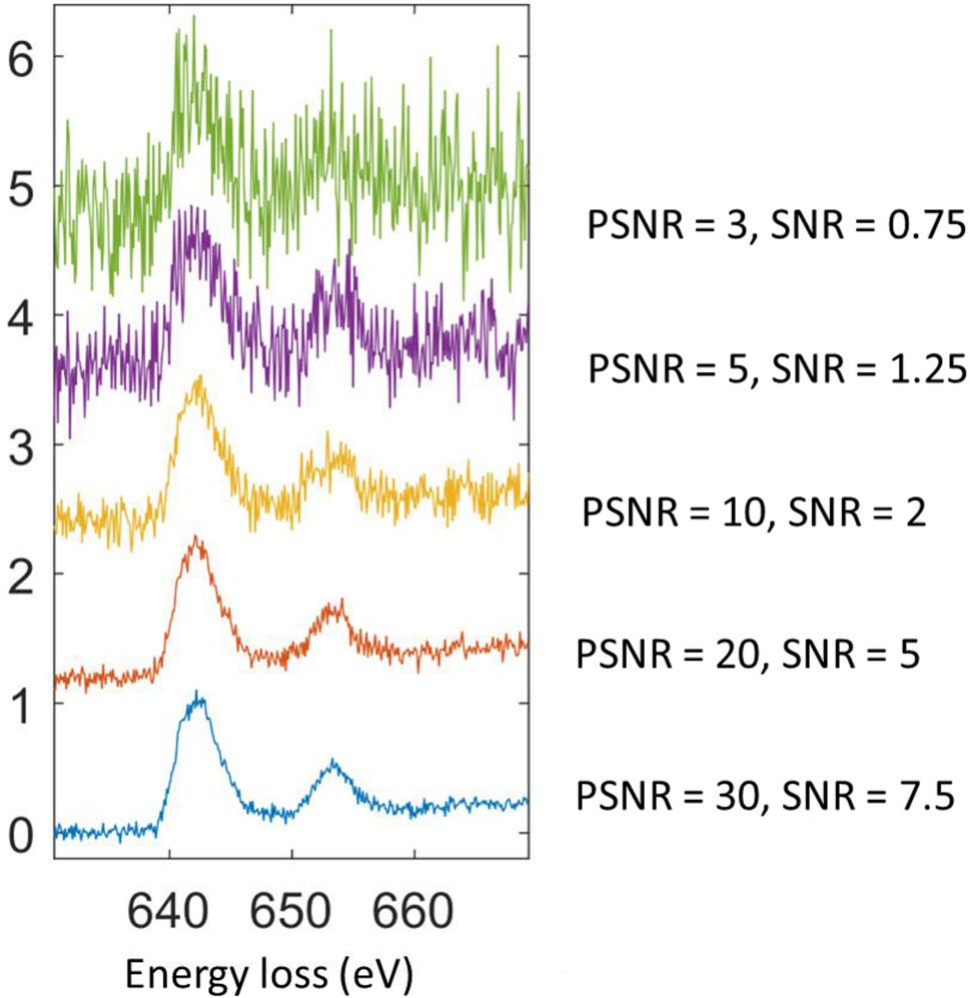
The Mn_3_O_4_ spectra from testing data as a function of noise.

The noise-contaminated Mn_3_O_4_ spectra are shown in Fig. 9. The predicted oxidation state decomposition is shown in Table 9. The ratio between 2+ and 3+ stays close to 1:2 for PSNR down to 10 (SNR down to 2). When PSNR is below 10 (SNR is below 2), the composition ratio starts to deviate from the theoretical ground truth as expected (the noise augmentation range is PSNR = [10,30]).

**Table 9:** Testing of CRN’s decomposition robustness against noise on testing data(Mn_3_O_4_).

PSNR	SNR	Predicted decomposition (%)	Predicted oxidation state
30	7.5	[29.41, 67.05, 3.54]	2.74
20	5	[36.23, 63.76, 0.01]	2.64
10	2	[38.01, 61.75, 0.24]	2.62
5	1.25	[47.8, 52.09, 0.1]	2.52
3	0.75	[66.91, 21.65, 11.45]	2.45

### Sensitivity and accuracy validation on Mn_3_O_4_ with vacancies on the tetrahedral sites

**Figure 10:**
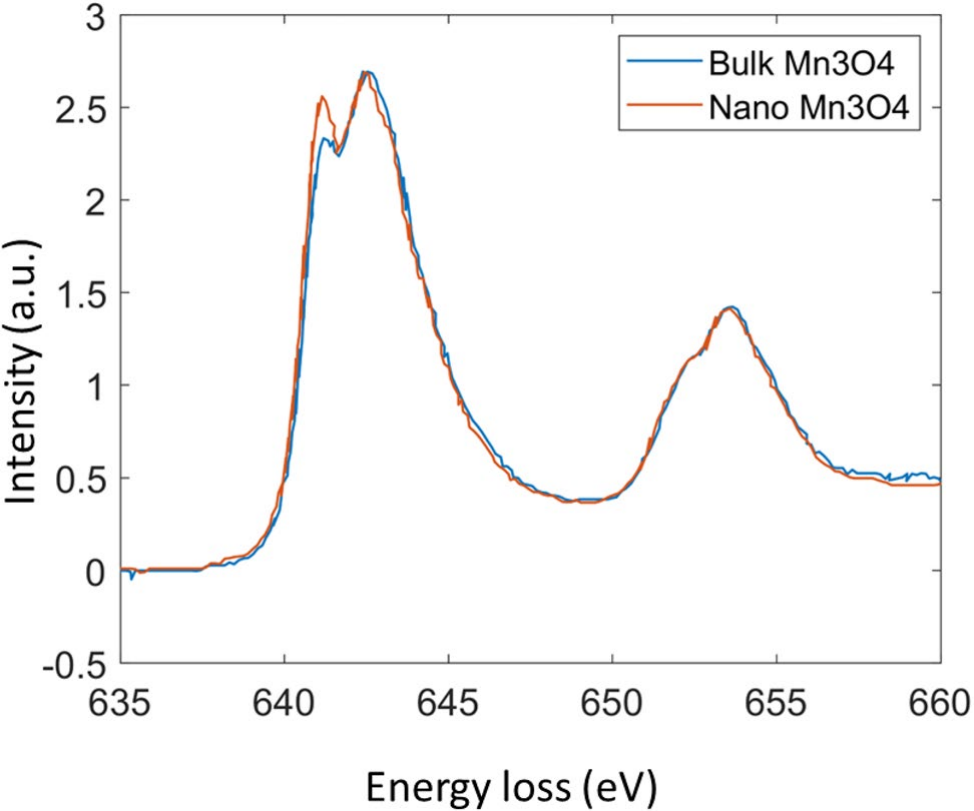
The EELS Mn L2,3 edges of bulk Mn_3_O_4_ vs nanosized Mn_3_O_4_.

We tested the accuracy and sensitivity of our model using two Mn_3_O_4_ EELS spectra documented in Ref.^9^. The two spectra are shown in Fig. 10. The predicted oxidation state decomposition is given in Table 10. The small difference in the L3 edge indicates that the nanosized Mn_3_O_4_ has slightly more Mn2+ and less Mn3+ than the bulk Mn_3_O_4_. The documented ratio of Mn3+/Mn2+ is 2 for the bulk sample and 1.6 for the nanosized sample in Ref.^9^. Our model accurately captures this change. For the nanosized sample, our model’s predicted decomposition clearly shows the reduction of Mn3+ composition and increase of Mn2+ composition. The ratio of Mn3+/Mn2+ is predicted to be 1.59 which is almost the same as the documented value. This test shows that our model is accurate and sensitive to small changes in the spectrum.

**Table 10:** Testing of CRN’s decomposition sensitivity on testing data(Mn_3_O_4_).

PSNR	Predicted decomposition (%)	Ratio documented in Ref.^9^ (%)
Bulk	[33.92, 64.33, 1.75]	[33, 67, 0]
Nano	[38.1, 60.51, 1.39]	[40, 60, 0]

### More on testing data

**Figure 11:**
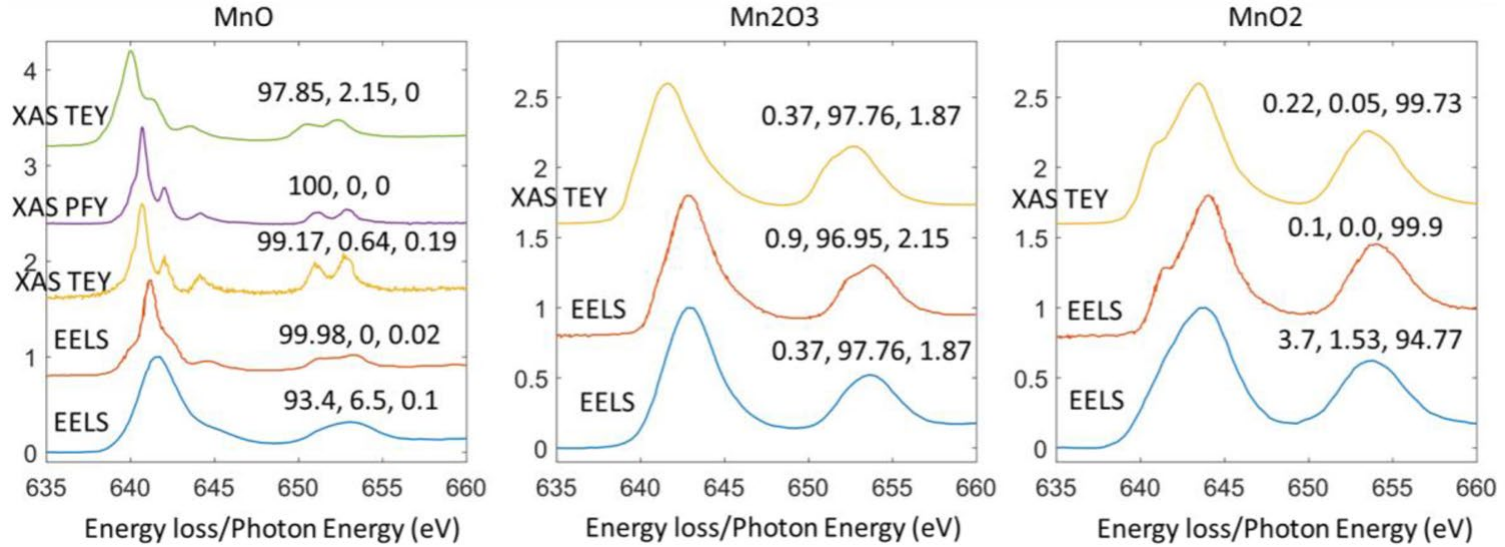
Validation of CRN’s decomposition sensitivity on data not used for training.

To further test the model on testing data. We collected more EELS and XAS data from literature and experiments with very different energy resolutions. All data shown in Fig. 11 were not used for training. The EELS data shown are from references^5, 19^ and the XAS data are from our own experimental collection at NSLSII and Taiwan Light Source. Figure 11 shows the oxidation decomposition of the EELS/XAS Mn L2,3 edge inferred by our model. All predictions are within reasonable errors of the ground truth. It is worth noting that the model is effective on both XAS and EELS spectra. The XAS and EELS have very different energy resolutions. Within the XAS, the TEY and PFY also have noticeable differences in fine structures. In addition, the energy onsets are all different. However, as shown, our model remains translation invariant and is robust enough to correctly decompose their oxidation states.

## Conclusion

In this work, we built a regression deep learning network to accurately decompose the mix valence state of Mn for both EELS and XAS spectra. By passing the Mn L2,3 edge spectra into the neural network, the ratio of Mn2+, 3+, and 4+ can be predicted. To train the network, we also created a forward model for synthesizing the mix valence state of Mn L2,3 edge spectra. Plural scattering, instrumentation broadening, noise, and energy axis offset were taken into account when creating the forward model. A 1.2 million spectral dataset was synthesized for network training and validation using the forward model. The network was also tested on the testing spectra, real spectra collected from experiments, which were not used in the dataset synthesis. The robustness of the network was examined against noise and plural scattering. The high accuracy of the network on both validation and testing spectra suggested it can accurately decompose Mn L2,3 edges. The robustness of the network against noise (PSNR down to 10) and plural scattering (t/λ up to 1) demonstrated the high sensitivity and stability of our network. Furthermore, the network performed quantitively well on common compounds such as MnO, Mn_2_O_3_, Mn_3_O_4_, and MnO_2_ which means our model can be deployed and trusted in real experiments. This work showed that it is possible to accurately decompose mix valence state of Mn L2,3 edge spectra for both EELS and XAS without reference and calibration using deep learning algorithms. In the future, the method described in this work can also be generalized to other transition metals such as Fe, since their similar chemistry property to Mn. This work provided a new angle to study the fine structure of L2,3 edges and the development of AI-driven autonomous TEM.

## Data Availability

Data and code are available from the corresponding author upon request.
